# Lipid and glucose metabolism in senescence

**DOI:** 10.3389/fnut.2023.1157352

**Published:** 2023-08-23

**Authors:** Bin Liu, Qingfei Meng, Xin Gao, Huihui Sun, Zhixiang Xu, Yishu Wang, Honglan Zhou

**Affiliations:** ^1^Department of Urology II, The First Hospital of Jilin University, Changchun, Jilin, China; ^2^Key Laboratory of Pathobiology, Ministry of Education, Jilin University, Changchun, Jilin, China

**Keywords:** senescence, lipid metabolism, glycolysis, CPT1, ACOX1, ACC, TCA, PPP

## Abstract

Senescence is an inevitable biological process. Disturbances in glucose and lipid metabolism are essential features of cellular senescence. Given the important roles of these types of metabolism, we review the evidence for how key metabolic enzymes influence senescence and how senescence-related secretory phenotypes, autophagy, apoptosis, insulin signaling pathways, and environmental factors modulate glucose and lipid homeostasis. We also discuss the metabolic alterations in abnormal senescence diseases and anti-cancer therapies that target senescence through metabolic interventions. Our work offers insights for developing pharmacological strategies to combat senescence and cancer.

## 1. Introduction

Senescence is a universal and complex biological process that affects all living organisms. While senescence contributes to embryonic development and limits tumorigenesis and tissue damage (1), it involves dynamic biological, environmental, physiological, psychological, behavioral, and social process changes leading to progressive declines in biological functions and physiological processes and increased susceptibility to disease, disability, and death (2, 3). The senescence process increases the risk of chronic diseases such as diabetic neurodegenerative diseases, metabolic syndrome, and cardiovascular disease, which have become more prevalent in the elderly population (4). Therefore, understanding the mechanisms and factors that influence senescence and its consequences is crucial for developing interventions to prevent, diagnose, treat, and delay age-related diseases and improve healthspan (the portion of life spent in good health).

Senescence and cell death are two different but interrelated phenomena. Cell death is the irreversible loss of cell viability and function that can occur through different mechanisms, including apoptosis, autophagy, necrosis, and senescence (5). In 2019, the International Cell Senescence Association reached a consensus on the characteristics and biomarkers of cellular senescence (6). They concluded that cellular senescence is not the same as cell death and that senescent cells remain metabolically active for some time and show distinct changes, with four characteristics typical of cell cycle arrest, senescence-related secretory phenotypes (SRSPs), macromolecular damage, and metabolic disorders. Senescent cells show reduced mitochondrial function, impaired adenosine triphosphate (ATP) production, and increased production of reactive oxygen species (ROS), which cause protein and lipid damage (macromolecular damage). Disorders in glucose and lipid metabolism are among the results and further drive the progression of senescence (7, 8).

Lipids may influence cell activity by altering cytomembrane composition, energy reserves, secondary messenger signaling, and gene expression (9–11). A common component of biological lipid species is fatty acids. One of the signs of senescence is fatty acid metabolic dysregulation, which is related to the transformation from catabolism to synthesis. A decline in fatty acid oxidation (FAO) capacity determines lipid imbalance and age-related obesity (12). Tissue-specific stem cells repair tissue damage and maintain tissue homeostasis. Unbalanced stem cell self-renewal can cause harmful effects such as disease and senescence. Age-related declines in FAO levels harm stem cell maintenance and activity, driving stem cell senescence (13). The imbalance between nuclear receptors (liver-X-receptor α, retinoid acid receptor α, and peroxisome proliferator-activated receptor α [PPARα]) and their target genes (ATP-binding cassette transporter A1, sterol regulatory element binding protein 1c [SREBP1c], and fatty acid synthase [FAS]) likely drives hepatic steatosis in aged patients (14). Moreover, H_2_O_2_-induced senescence is significantly decreased by inhibiting lipogenesis using FAS inhibitors or silencing SREBP1 and ATP citrate lyase (15). All these pieces of evidence indicate that fatty acid metabolism seriously affects senescence. Targeting the key enzymes of fatty acid metabolism is the most direct and scientific means to screen for anti-senescence drugs or promote tumor senescence treatment.

Glucose metabolism is also involved in the regulation of intracellular senescence. Glucose transporters (GLUTs), members of the membrane transporter family, transport extracellular glucose into the cell. GLUT1 is highly expressed in senescent cells and increases glucose uptake (16, 17). In contrast, under low-concentration glucose culture conditions, drug-induced senescence in lung cancer cells (17) or replicative senescence of human fibroblasts (18) and stem cells (19) cultured *in vitro* is delayed (20). Glucose transported into cells by GLUTs is catalyzed to pyruvate via glycolysis enzymes. The levels of multiple glycolytic enzymes, including hexokinases (HKs), lactate dehydrogenase A (LDHA), and pyruvate kinase (PK), are increased in senescent cells (21). Finally, the metabolites generated by pyruvate entering the tricarboxylic acid (TCA) cycle, including α-ketoglutaric acid (αKG) (22–24) and citrate (25, 26), significantly inhibit intracellular senescence.

This review aims to clarify the relationship between lipids, glucose metabolism, and senescence.

## 2. Lipids and senescence

### 2.1. Fatty acid catabolism and senescence

Fatty acid catabolism includes mitochondrial β-oxidation of saturated fatty acids, peroxisomal β-oxidation of very-long-chain fatty acids, α-oxidation of branched-chain fatty acids, ω-oxidation, and ketone formation (27–31). Mitochondrial β-oxidation of saturated fatty acids and peroxisomal β-oxidation of very-long-chain fatty acids are generally the primary metabolic pathways, and carnitine palmitoyl transferase 1 (CPT1) and acyl-CoA oxidase 1 (ACOX1) play crucial roles as rate-limiting enzymes.

#### 2.1.1. Mitochondrial β-oxidation

Under sufficient oxygen supply, medium- and long-chain fatty acids can be decomposed into acetyl-CoA, which can be further completely oxidized into CO_2_, H_2_O, and significant energy. The whole process of converting acyl CoA to acetyl-CoA, called mitochondrial β-FAO, occurs in the mitochondrial matrix (32). CPT1 assists acyl CoA in the transmembrane entry into the mitochondria and is the primary rate-limiting enzyme (33). All three isoforms of CPT1 (CPT1A, CPT1B, and CPT1C) are associated with senescence. Comparison with proteomic analysis of adult mouse kidneys revealed significantly lower CPT1A expression in aged mice (34). In diabetic nephropathy, a common disease associated with senescence, CPT1A expression is similarly suppressed (35). The same trend in CPT1B expression has been observed in aged rats (36). In addition, deletion of the novel biomarker CPT1C induced mitochondrial dysfunction, leading to senescence-like growth inhibition and cellular senescence in six cancer cell types, including pancreatic cancer cells, and inhibited cancer growth *in vivo* (37). While these findings suggest that senescence is inextricably linked to the absence of CPT1, the exact mechanism is not yet clear.

**Figure 1:**
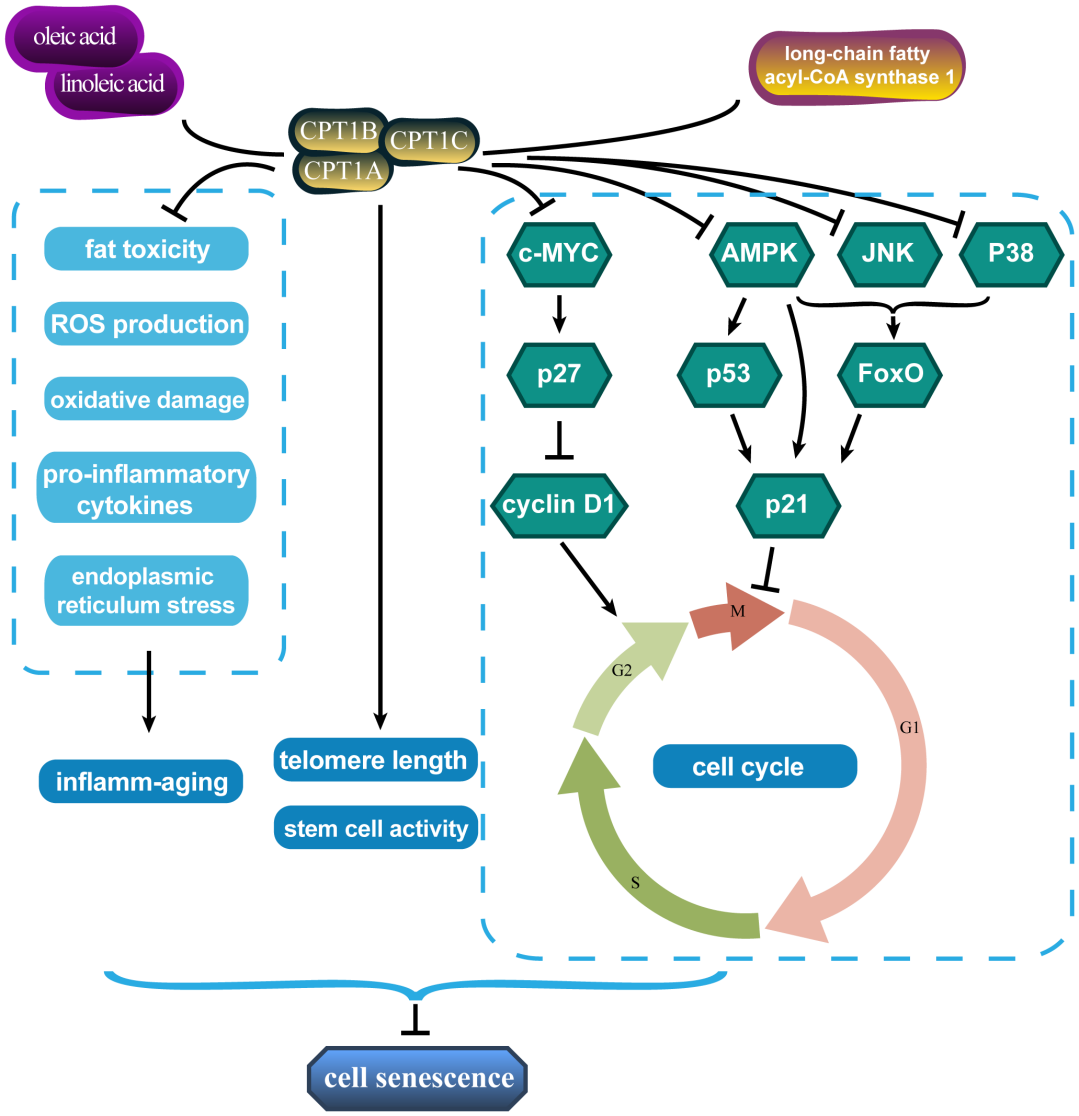
Carnitine palmitoyl transferases affect senescence through multiple pathways. CPT1, carnitine palmitoyl transferase 1.

In recent years, scholars have made progress in this regard (Figure 1). Telomeres, the protective caps on the ends of chromosomes, guard genetic material during cell division. Telomeres shorten with each cell division until they reach a critical length that triggers cellular senescence or apoptosis (38). Telomere shortening is one of the hallmarks of senescence, as it reflects the cumulative damage and stress that cells experience over time. In human hepatoma (HepG2) cells, the knockdown of patatin-like phospholipase domain-containing 2 (also called adipose triglyceride lipase [ATGL]) and CPT1, two crucial genes for lipolysis, results in reduced telomere length (39).

In addition, the cell cycle regulates cell differentiation, proliferation, and senescence. Moreover, senescent cells are blocked in certain growth phases. Treatment with linoleic and oleic acids significantly increased CPT1 expression and S-phase ratio in primary bovine satellite cells and promoted cell proliferation, while decreasing the G0/G1 phase ratio (40). Long-chain fatty acyl-CoA synthase 1, which catalyzes the bioconversion of exogenous or *de novo* synthesized fatty acids to fatty acyl-CoA, promoted tumor progression by reducing CPT1 activity and the number of prostate cancer cells remaining in the G1/S phase (41). The triggers of senescence ultimately converge on the p53/p21^CIP1^ and p16^INK4A^/pRb pathways, which induce cell cycle arrest (42, 43). The proto-oncogene c-MYC is involved in cell cycle progression, apoptosis, and cellular transformation. CPT depletion, especially CPT1C, leads to cellular senescence by blocking the cell proliferation-promoting and cell cycle progression effects of the c-MYC/p27/cyclin D1 signaling axis (44). CPT1 overexpression increases ATP production from FAO sources and drives cell cycle operation in pulmonary artery smooth muscle cells by inhibiting the adenosine 5′-monophosphate (AMP)-activated protein kinase (AMPK)-p53-p21 signaling pathway (45). CPT1A also represses p21 expression by inhibiting forkhead box O (FoxO), which must be phosphorylated by various protein kinases such as AMPK, c-Jun N-terminal protein kinase (JNK), and p38 to perform their functions (46).

The loss of stem cell number and function is also a visual manifestation of senescence. This age-related change underlays some adverse consequences of senescence. Mihaylova et al. (47) showed that interference with CPT1A reversed fasting-induced enhancement of intestinal stem cell activity and that CPT1A deficiency reduced ISC number and function. Moreover, CPT1A-mediated FAO facilitates the maintenance of neural stem/progenitor cell quiescence (48, 49). Chronic inflammation is a defining characteristic of senescence and has been dubbed “inflamm-senescence” (50). Mitochondrial FAO with CPT1 drives anti-inflammatory protective effects due to its ability to remove pro-inflammatory compounds such as saturated fatty acids by reducing fat toxicity and ROS production and attenuating palmitic acid-induced pro-inflammatory cytokines, oxidative damage, and endoplasmic reticulum stress (51, 52).

In conclusion, these results tentatively explain the anti-senescence effects of mitochondrial β-oxidation and CPT1. Targeted increase or inhibition of CPT1 expression for different diseases may reduce age-related senescence damage or inhibit tumor progression.

#### 2.1.2. Peroxisomal β-oxidation

Mitochondrial β-oxidation metabolizes common saturated fatty acids. However, some fatty acids, such as very long-chain fatty acids (VLCFAs, with >22 carbons), require the peroxisomal β-oxidation-dependent pathway to be converted into chain-shortened acyl-CoA, which is then transported to the mitochondria for complete oxidation (53). ACOX1 catalyzes its rate-limiting step. Numerous senescence-related diseases are directly related to fatty acid peroxisome β-oxidation. Age-related reduction in liver peroxisomal β-oxidation was accompanied by altered brain fatty acid composition and decreased ACOX1 and PPARα expression in old rats (54). In senescence and age-related degenerative diseases, peroxisome β-oxidation and ACOX1 are significantly reduced (55). In the early stages of Alzheimer’s disease (AD), ACOX1 and its transcriptional regulators PPARα and peroxisome proliferator-activated receptor gamma coactivator 1 α (PGC-1α) are induced to be highly expressed due to damaged mitochondria and increased oxidative stress but are significantly reduced in late stages (56). Moreover, decreased ACOX1, CPT1, and PPARα expression in leptin-resistant elderly rats is the basis of age- and lipid-related diseases (57).

**Figure 2:**
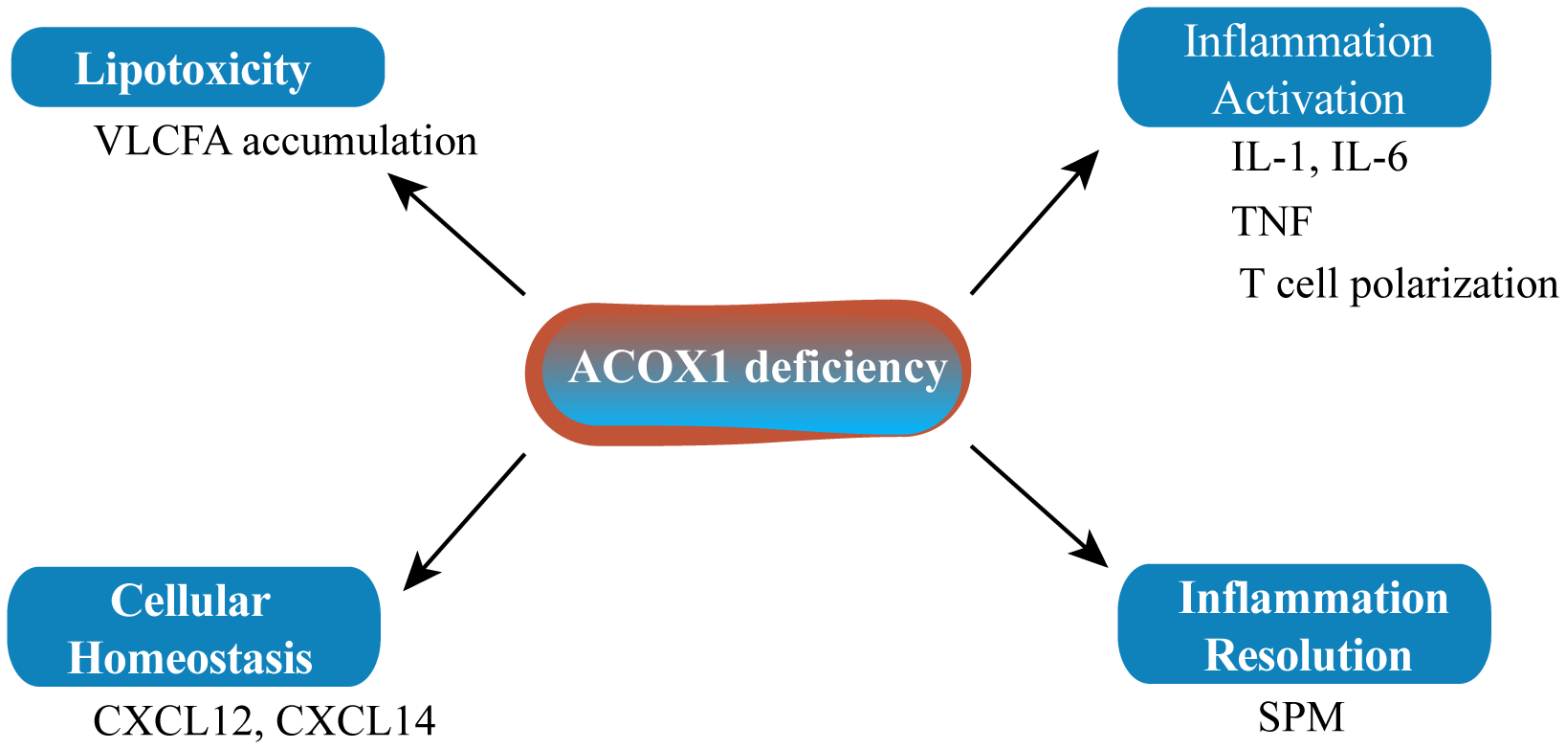
ACOX1 deletion affects cellular homeostasis. SPM, specialized pro-resolving mediator.

ACOX1 deficiency leading to the accumulation of specific metabolites may affect the biogenesis of the peroxisome itself and/or the homeostasis of other cellular organelles such as the mitochondria and endoplasmic reticulum (Figure 2). Inflammation plays an important role in senescence. The main characteristics of pseudo-neonatal adrenoleukodystrophy (a neurodegenerative disease) are VLCFA accumulation and inflammatory demyelination due to activation of the IL-1 inflammatory pathway. The main cause of this lesion is the absence of ACOX1 (58). Fibroblasts from an ACOX1-deficient patient showed downregulation of numerous cytokines and chemokine mRNAs, including CXCL14 and CXCL12, which are relevant to the regulation of cellular homeostasis (58). Mice lacking ACOX1 also exhibited elevated levels of inflammatory cytokines such as interleukin-6 (IL-6) and tumor necrosis factor-α (TNF-α), and induced T cell polarization (59). However, inflammation resolution is a dynamic biosynthetically active process governed by a superfamily of bioactive lipids known as specialized pro-resolving mediators (SPMs), which enhance the remission of inflammatory reactions (60). SPMs can regulate autophagy, change the release of cytokines/chemokines, and affect the migration and function of leukocytes to alleviate inflammation and delay the senescence process. As it is upstream of SPM biosynthesis, ACOX1 has become a potential anti-senescence protein (61, 62).

PPARα, a subtype of PPARs (including α, β/δ, and γ) and a transcriptional activator of *ACOX1* and *CPT1*, regulates the fatty acid β-oxidation pathway (63, 64). PPARα activity and expression decreased in various aged-animal organs, including the liver and heart (65, 66). These reductions are most likely directly connected to lipid metabolic abnormalities and elevated levels of inflammatory mediators in elderly animals (67). Fatty acid-binding protein-1 (FABP1) binds to free fatty acids by upregulating PPARα and protects the liver from fat toxicity (68). Senescence decreases FABP1 levels, which alters PPARα and causes β-oxidation damage, increasing the morbidity of non-alcoholic fatty liver disease (69). PPARα depletion inhibits cell proliferation and induces cell senescence by reducing the expression of the target gene *CPT1C* (70).

Drugs targeting PPARα may have the potential to fight age-related diseases such as atherosclerosis, vascular diseases, and AD (71). The receptor for advanced glycation end product (RAGE) plays a crucial role in senescence-related vascular disorders, liver damage, and insulin resistance (72–74). Wan et al. (75) observed elevated RAGE levels at the molecular level, which was associated with lower PPARα and β-oxidation levels and was responsible for the deposition of hepatic TG in aged mice. Thus, the RAGE/PPARα regulator axis may be a therapeutic target for fatty liver disease associated with senescence. In *Caenorhabditis elegans*, the administration of ω-3 polyunsaturated fatty acids (α-linolenic acid) increased longevity by activating the transcription factors NHR-49/PPARα (76). Oleoylethanolamide (OEA) can interact with hepatocyte nuclear factor 4 and PPARα and promote their transcriptional activity. Worms treated with OEA showed PPARα activation and increased lifespans (77).

Fenofibrate, a PPARα agonist, reduces the number of senescent cells by increasing apoptosis and autophagy flux and prevents cartilage degeneration caused by senescent and osteoarthritis (78). Proanthocyanidins correct lipid metabolism disorders and treat senescence-related diseases by regulating key enzymes, including PPARα and CPT1 (79). In a d-galactose-induced senescent mouse model, mussel peptides mitigated lipid metabolic diseases and the senescence phenotype by preserving glucose and lipid homeostasis and increasing PPARα/PPARγ expression levels (80). These findings suggest that PPARs may be essential targets in lipid signaling pathways that promote lifespan. Age-related modifications in PPARα in humans have not been explicitly proven, and determining whether the results of animal experiments are applicable to humans is difficult. However, these results provide molecular support for the anti-senescence application of PPAR agonists (e.g., fibrates).

#### 2.1.3. Fatty acid α-/ω-oxidation

Fatty acid α-oxidation occurs in the peroxisomes of cells. This process involves the degradation of branched-chain fatty acids, such as phytanic acid, produced by the body or obtained through dietary intake. Phytanic acid is a 20-carbon fatty acid with a methyl group at the γ-carbon, which prevents it from undergoing β-oxidation (81). Therefore, phytanic acid is oxidized at the α-carbon, which is adjacent to the carboxyl group and loses one carbon atom as CO_2_. Phytanoyl-CoA hydroxylase is involved in this process. The resulting product, pristanic acid, can then enter the β-oxidation pathway and generate acetyl-CoA and propionyl-CoA. Fatty acid α-oxidation and senescence are related in several aspects. First, fatty acid α-oxidation is impaired in some senescence-related diseases, such as Refsum disease, a rare genetic disorder that causes the accumulation of phytanic acid in various tissues and plasma, leading to neurological symptoms such as peripheral neuropathy, ataxia, retinitis pigmentosa, and deafness (82). Second, fatty acid α-oxidation generates ROS as by-products (83), which can cause oxidative stress and damage to cellular components, including DNA, proteins, and lipids. Oxidative stress is a major contributor to senescence and age-related diseases such as neurodegeneration, cancer, and diabetes. Third, α-oxidation of fatty acids is also regulated by PPARs (84), the latter in relation to senescence as discussed above regarding peroxisomal β-oxidation.

Fatty acid ω-oxidation occurs in the endoplasmic reticulum of some cells, especially in the liver and kidney (29). This process involves the monooxygenase-catalyzed oxidation of the fatty acid ω-carbon (the carbon furthest from the carboxyl group), followed by successive oxidations of the β-carbon until the fatty acid chain is shortened by two carbon atoms. This mechanism exists to break down large, water-insoluble fatty acids that, in greater quantities, would be hazardous to cells (85). The ω-oxidation of fatty acids accelerates the rate of fatty acid degradation as feedback to various stressors such as hypoxia, inflammation, and stimulation by exogenous substances. These substances increase the demand for FAO and detoxification. Correspondingly, the omega-oxidation of fatty acids reduces the likelihood that these stressors will lead to senescence and age-related diseases.

### 2.2. Fatty acid synthesis and senescence

Triglycerides, phospholipids, sphingolipids, and cholesterol lipids are all produced by a series of enzymatic reactions between fatty acids and different chemical groups. Therefore, the regulation of fatty acid synthesis affects the metabolic homeostasis of lipids to a certain extent.

#### 2.2.1. Acetyl-CoA carboxylase

Acetyl-CoA carboxylase (ACC) catalyzes the rate-limiting step of *de novo* fatty acid synthesis (86). Among the two subtypes, ACC1 and ACC2, the former locates in the cytoplasm and is used for fatty acid synthesis by converting acetyl coenzyme A into malonyl coenzyme A. ACC2 is located on the cytoplasmic surface of mitochondria, and the product is used to inhibit CPT1 from reducing FAO (87). SiRNA silencing of SREBP1 significantly reduced the expression of its downstream target genes *ACC*, *FAS*, and ATP citrate lyase, and weakened H_2_O_2_-induced senescence (15). ACC1-dependent lipogenesis is the fundamental metabolic pathway downstream of AMPK, which induces autophagy and maintains cell survival during yeast senescence (88). This evidence suggests that ACC directly modulates the senescence phenotype, and several drugs targeting ACC to slow senescence already exist. Citrate treatment can increase ACC1 expression, promote excessive lipid biosynthesis, and lead to tumor cell senescence and growth inhibition (26). The AD candidate drugs CMS121 and J147 play anti-senescence neuroprotective roles by inhibiting ACC1 and increasing acetyl-CoA levels (89). Resveratrol plays an anti-senescence role by increasing ACC phosphorylation and enhancing mitochondrial lipolysis ability (90). AMPK, the upstream negative regulator of ACC, also shows some anti-senescence activity.

#### 2.2.2. AMP-activated protein kinase

Lipid metabolism depends heavily on AMPK. AMPK enhances lipolysis by upregulating ATGL expression (91). AMPK can also suppress lipid production by inhibiting ACC activation by phosphorylating ACC1 (Ser 79 Ala) and ACC2 (Ser 212 Ala) (92) and downregulating SREBP1c expression (93, 94). AMPK affects organism senescence directly or indirectly in a variety of ways. For example, dietary restrictions delay the senescence of many species, in which AMPK is widely involved. In *C. elegans*, AMPK and dietary restriction increase FAO through mitochondrial-peroxisome coordination, thereby maintaining homeostasis and plasticity within the mitochondrial network to extend life (95). Greer et al. (96) also reported that the lifetime extension due to food restriction in *C. elegans* was mediated through the AMPK-FoxO (transcription factors) pathway. AMPKα subunit AAK-2 is activated by either a mutation that reduces insulin-like signaling or an environmental stressor that increases the AMP:ATP ratio, which can extend lifespan *in C. elegans* (97, 98). A similar situation exists in mice. For instance, the pharmacological stimulation of AMPK can imitate caloric restriction (induced comparable gene expression patterns) and provide extensive protection against age-related diseases (99). Fibroblast growth factor 21 can exhibit anti-senescence effects by elevating the expression of AMPK, which regulates lipid and glucose metabolic balance (100), blocking the p53 signaling pathway in an AMPK-dependent manner (101). As an AMPK agonist, metformin plays a dual role in cancer prevention and anti-senescence (102, 103). Fruit flies’ lifespans also increased by overexpressing AMPK (104).

AMPK also regulates the nuclear factor kappa-light-chain-enhancer of activated B cells (NF-κB) inflammatory pathway (105), mammalian target of rapamycin (mTOR) C1 autophagy-related protein, and PGC-1α, P53/HIF-1α senescence-related signaling to achieve anti-senescence effects (106, 107). In detail, by phosphorylating mTORC1, ULK1, and PIK3C3/VPS34 complexes, AMPK directly stimulates autophagy (108). Inhibiting mTOR signaling plays a crucial role in mammalian senescence by reducing the accumulation of protein toxicity and oxidative stress (99), increasing autophagy to clear damaged proteins and organelles (109), and enhancing the self-renewal capacity of hematopoietic stem cells (110) and intestinal stem cells (111). Metformin can reduce cartilage degeneration in senescence-related osteoarthritis models by modulating AMPK/mTOR (112).

### 2.3. Ketone bodies and senescence

Ketone bodies are endogenous metabolites produced by the liver from fatty acids and ketogenic amino acids when glucose availability is low, such as during fasting, caloric restriction, or prolonged exercise (113). Ketone bodies can be used as an alternative fuel source by many tissues, especially the brain, heart, and skeletal muscle, when glucose is scarce or absent (114). These bodies can also cross the blood–brain barrier and supply energy to the central nervous system.

Ketone bodies are scary to clinicians because of the high mortality rate of ketoacidosis; however, in reality, the ketone bodies have signaling functions that regulate inflammation, epigenetics, oxidative stress, and other cellular processes by binding to specific receptors and enzymes (115–117). The bodies may also modulate gene expression and protein synthesis. Ketone bodies are linked to multiple mechanisms of senescence and resilience, such as glucose sparing, mitochondrial biogenesis, autophagy, and hormesis (118–121); thus, they have anti-cancer, anti-angiogenic, and anti-atherogenic effects. Ketone bodies also reduce neuroinflammation and β-amyloid and tau accumulation, and improve memory and healthy lifespan in a senescent mouse model (114).

The most well-known ketone body, 3-hydroxybutyrate (3-OHB), binds to specific hydroxycarboxylic acid receptors to inhibit histone deacetylases, free fatty acid receptors, and nucleotide oligomerization domain (NOD)-like receptor protein 3 inflammasomes. This initially inhibits lipolysis, inflammation, oxidative stress, cancer growth, angiogenesis, and atherosclerosis, and may increase lifespan associated with exercise and caloric restriction (122). In addition to helping treat other neurological conditions including dementia, the ketone body/ketogenic diet has been successfully utilized to reduce seizures (123). Additionally, 3-OHB protects muscle proteins from damage caused by systemic inflammation and is a crucial part of the metabolic defense against insulin-induced hypoglycemia (124).

### 2.4. Phospholipids and senescence

Phospholipids are major components of biological membranes and consist of a glycerol backbone, two fatty acid chains, and a polar head group. Phospholipids are essential for membrane structure, fluidity, and function, as well as for intracellular signaling, trafficking, and metabolism (125). Phospholipids are synthesized in different cellular compartments by various enzymes and transported by specific carriers or vesicles. Phospholipids mainly include phosphatidylcholine, phosphatidylethanolamine, phosphatidylserine, phosphatidylinositol, phosphatidic acid, and cardiolipin. Phospholipids can also be degraded or modified by phospholipases, acyltransferases, and other enzymes that regulate their turnover and diversity (126).

Phospholipids play important roles in senescence and age-related diseases. Senescence is associated with changes in phospholipid composition, metabolism, and transport in different tissues and organs. These changes may affect membrane integrity, fluidity, and function, as well as cellular signaling and homeostasis (127, 128). For example, senescence leads to decreased unsaturated fatty acid levels and increased saturated fatty acid levels in phospholipids, which may impair membrane fluidity and increase oxidative stress (129). Senescence also alters the levels of specific phospholipid species, such as phosphatidylcholine, phosphatidylethanolamine, and cardiolipin, which may affect mitochondrial function and biogenesis (130).

Phospholipid interventions may have beneficial effects on senescence and lifespan. Dietary supplementation or genetic manipulation of phospholipids or their precursors can modulate membrane properties, cellular signaling, and mitochondrial function in various model organisms. For instance, phosphatidylcholine or choline supplementation improved cognitive function and memory in aged rodents (131). Overexpression of cardiolipin synthase or supplementation of cardiolipin precursors can enhance mitochondrial function and extend lifespan in yeast, worms, flies, and mice (132).

### 2.5. Sphingolipids and senescence

Sphingolipids are a diverse class of lipids that are involved in various cellular processes such as membrane structure, signal transduction, cell cycle regulation, apoptosis, senescence, and inflammation (133). Sphingolipids are synthesized *de novo* in the endoplasmic reticulum from non-sphingolipid precursors, such as serine and palmitoyl-CoA, and then further modified in the Golgi apparatus and other organelles to generate a variety of complex sphingolipids with different polar head groups (134). The major bioactive sphingolipids include ceramide (Cer), sphingosine, sphingosine-1-phosphate (S1P), and ceramide-1-phosphate, which can act as second messengers or ligands for specific receptors to modulate cellular responses (135).

Sphingolipids have been implicated in the regulation of senescence and age-related diseases, as they can affect several hallmarks of senescence such as genomic instability, telomere attrition, epigenetic alterations, loss of proteostasis, deregulated nutrient sensing, mitochondrial dysfunction, cellular senescence, stem cell exhaustion, and altered intercellular communication (136). In general, Cer and its derivatives induce cellular senescence and promote senescence phenotypes, while S1P and its receptor signaling delay senescence and extend lifespan (137). For example, sphingolipids are involved in sarcopenia, an age-related disorder of loss of skeletal muscle mass and function. Park et al. (133) recently reported that Cer and glucosylceramide accumulate in the skeletal muscle of a senescent mouse model and that deleting serine palmitoyltransferase (SPT), the rate-limiting enzyme for sphingolipid biosynthesis, or using the SPT inhibitor myriocin to inhibit sphingolipid synthesis prevents sarcopenia and improves muscle health. In addition, SPT deletion or myriocin treatment enhances mitochondrial function, autophagy, and proteostasis in aged muscle cells, suggesting that sphingolipids may impair these processes by affecting endoplasmic reticulum stress and calcium homeostasis.

### 2.6. Sterols and senescence

Sterols are essential components of eukaryotic cell membranes, where they regulate membrane fluidity, permeability, and microdomain formation. Sterols also have important roles in animal physiology, as they are precursors of steroid hormones such as glucocorticoids, mineralocorticoids, androgens, estrogens, and progestins, which modulate various metabolic, reproductive, and immune functions (138). Sterols are synthesized from acetyl-CoA through a complex pathway that involves more than 20 enzymes and intermediates (139). The first steps of sterol biosynthesis occur in the cytosol and endoplasmic reticulum, where acetyl-CoA is converted to mevalonate by three enzymes: acetoacetyl-CoA thiolase, HMG-CoA synthase, and HMG-CoA reductase. Mevalonate is then converted to isopentenyl pyrophosphate (IPP) and dimethylallyl pyrophosphate (DMAPP), which are the building blocks of isoprenoids. IPP and DMAPP are used to synthesize geranyl pyrophosphate, farnesyl pyrophosphate, and squalene, which are the precursors of sterols and other isoprenoids such as ubiquinone, dolichol, and prenylated proteins (140). The final steps of sterol biosynthesis involve the cyclization of squalene to form lanosterol, which is then converted to cholesterol by a series of modifications such as hydroxylation, demethylation, reduction, and isomerization. Take cholesterol, the predominant sterol in animals, for example, which is integral and indispensable to neuronal physiology during both development and adulthood. As a major component of cell membranes and a precursor to steroid hormones, it helps regulate ion permeability, cell shape, intercellular interactions, and transmembrane signaling. Inherited diseases with mutations in cholesterol-related genes lead to impaired brain function. In these cases, brain cholesterol defects may be secondary to pathogenic factors and lead to functional deficits through altered synaptic function. Defects in brain cholesterol metabolism may lead to neurological syndromes such as AD, Huntington’s disease, and Parkinson’s disease, and even cognitive deficits (141–143).

## 3. Glucose metabolism and senescence

### 3.1. Glycolysis

The Warburg effect reveals that tumor cells prefer ATP production by glycolysis over oxidative phosphorylation even in the presence of abundant oxygen (144). The Warburg effect plays an important role in the inhibition of cellular senescence and tumor promotion (145). Glycolysis is upregulated in most senescence phenotypes. Glycolytic gene overexpression promotes altered cancer metabolism in a variety of tumor cells. LDHA, involved in the conversion of pyruvate to lactate, was significantly increased in radiotherapy-induced senescent HCT-116 and MDA-MB-231 cells (146, 147). Lactate liberation acidulates the extracellular environment involved in SRSPs (146). Lactic acid-induced acidic intracellular pH activates Snail to promote epithelial-mesenchymal transition. Increased Snail expression facilitated lung cancer cells to escape oncogene-induced senescence by directly suppressing p16^INK4a^ expression (21). In this way, LDHA expression in senescent fibroblasts promotes PC3 cell invasion in the co-culture of senescent fibroblasts and PC3 cells (148). Pharmacological inhibition of LDHA induces tumor cell senescence by suppressing heat shock response (149). Aurora-A, a serine/threonine kinase, can directly bind to the transcription factor SOX8 to promote the expression of glycolytic and senescence genes, including *LDHA*, *HK2*, *P16*, and *hTERT* in cisplatin-resistant ovarian cancer cells (150). Consequently, radiotherapy/chemotherapy-induced senescent cancer cells exhibit glycolytic vulnerability.

Beyond radiotherapy- and chemotherapy-induced senescent cancer cells, glycolysis is also activated in non-neoplastic senescent cells. The levels of intermediate metabolites of glycolysis, including 3-phosphoglycerate, glucose 6-phosphate, fructose 6-phosphate, and phosphoenolpyruvate, are significantly increased in senescent fibroblasts, which highlights increased glycolytic flux to promote pyruvate and lactate production (151). Senescent spermatogonial stem cells exhibit JNK phosphorylation and enhanced glycolytic capacity compared with young cells (152). Senescence fibroblasts exhibit a significant increase in glucose consumption (18). Increased glycolysis appears to be mediated by the overexpression of multiple glycolytic enzymes in distinct kinds of induced senescence. First, GLUT proteins are encoded by *SLC2*. GLUTs 1–4 have widely established roles as glucose transporters (153). GLUT1 is overexpressed in aged lungs and regulates fibrogenesis (154). In this way, hyperglycemia-induced GLUT1 overexpression activates the mTOR pathway to increase P16 and P21 levels while promoting macrophage establishment of the SRSP response (16). Second, HKs catalyze the phosphorylation of glucose as the first rate-limiting step in glycolysis (155). HK1 and HK2 (154) isozymes are highly expressed in replicative senescence human fibroblasts (18). Third, PK, the rate-limiting enzyme in the final step of glycolysis, catalyzes pyruvate production. The levels of this enzyme are upregulated in replicative senescence fibroblasts due to increased TCA activity and oxygen consumption (156). In conclusion, glycolysis is increased in replicative senescent cells, but the regulatory mechanism of elevated enzyme expression and activity needs to be further characterized.

### 3.2. TCA cycle

Tumor cells maintain cell proliferation by activating glycolysis to provide energy and precursors. Pyruvate, a glycolytic terminal product, enters the TCA cycle to produce metabolic intermediates that inhibit cellular senescence (157). Citrate is the product catalyzed by citrate synthase in the initiation of the TCA cycle. Recently, citrate has been reported to extend lifespan through mTOR and AMPK. However, citrate treatment results in cellular senescence and inhibits the proliferation of MCF-7 and HCT116 cancer cells (26). Mechanistically, DNA damage pathways coupled with MAPK and mTOR pathways lead to citrate-induced cellular senescence (26). αKG, a metabolite catalyzed by isocitrate dehydrogenase (IDH), extends the lifespan of *C. elegans* (23), *Drosophila* (158), and mice (22); attenuates mouse age-related bone loss (24); and delays age-related fertility decline in mammals (159). Sustaining this biological activity requires a continuous supply of ATP from mitochondria; however, the partial inhibition of the electron transport chain by αKG reportedly extends the lifespan of *C. elegans* and mammalian cells (23). Mechanistically, αKG inhibits intracellular senescence by regulating the expression of histone epigenetic modifications of BMP signaling proteins (24). In addition, αKG prolongs *Drosophila* lifespan by activating AMPK and inhibiting mTOR (158). The IDH1 R132H mutant indirectly promotes senescence of malignant glioma cells by reducing αKG production (160).

### 3.3. Pentose phosphate pathway

Abnormal glucose metabolism in cancer cells activates arrested cellular senescence. Cancer cells not only show active glycolysis for sufficient energy but also precursors from the pentose phosphate pathway (PPP) for intracellular biosynthesis (161). G6PD-mediated oxidative PPP (oxPPP) provides NADPH to counteract oxidative stress, while non-oxPPP generates ribose-5-phosphate (R5P) to provide precursors for nucleotide synthesis (161). G6PD, a unique rate-limiting enzyme in the PPP, is involved in counteracting cellular senescence. Mechanistically, G6PD knockdown increases the levels of P21, a classical marker of senescence, which promotes HCC and HCT116 cell senescence (162). In addition, TAp73 enhances cell cycle inhibitory protein P21 by regulating metabolism (163). TAp73 directly activates G6PD to increase PPP flow to inhibit cancer cell senescence (164). Thus, G6PD may mediate, at least in part, the regulation of the senescence phenotype of cancer cells by TAp73. G6PD also regulates cellular senescence by affecting telomerase activity (165, 166). G6PD-deficient fibroblasts exhibit delayed growth and accelerated senescence. Ectopic expression of the human telomerase reverse transcriptase hTERT activated telomerase activity to prevent cellular senescence in G6PD-deficient fibroblasts (166). Thus, the knockdown of hTERT significantly reduces G6PD expression and telomerase activity to promote cancer cell senescence (165). 6-phosphogluconate dehydrogenase (6PGD) is a key enzyme in oxPPP. Pharmacological inhibition of 6PGD induces cellular senescence and MCF-7 cell cycle arrest (167). Non-oxPPP also regulates cellular senescence. Supplementation of ribose 5-phosphate in drug-induced senescent human dermal fibroblasts significantly inhibited cell enlargement, a morphological alteration of cellular senescence (168). In addition, hTERT knockdown inhibited the expression and activity of transketolase, a key enzyme of non-oxppp; increased cancer cell senescence; and reduced tumor burden in a mouse model of heterotypic xenograft (165).

## 4. Lipid and glucose metabolism and SRSPs

SRSPs describe the diverse array of proinflammatory and profibrotic factors secreted by senescent cells (169). SRSPs include cytokines, chemokines, growth factors, proteases, and extracellular matrix components that can affect the surrounding tissue microenvironment and modulate various biological processes, including inflammation, immunity, tissue remodeling, and tumorigenesis (170). SRSPs are considered crucial drivers of chronic inflammation and senescence phenotypes, as they accumulate throughout normal senescence and in age-related diseases. SRSPs can have both beneficial and detrimental effects on the organism, depending on the expression context and duration. While SRSPs can promote wound healing, tissue repair, and immune surveillance by stimulating cell proliferation, angiogenesis, and immune cell recruitment (170), they can also induce cellular senescence in neighboring cells, disrupt tissue homeostasis and function, and facilitate the development and progression of age-related diseases such as cancer, neurodegeneration, cardiovascular disease, diabetes, and osteoporosis (171, 172).

Growing evidence has shown that lipid metabolism and SRSPs are interconnected at multiple levels. For instance, some lipids (such as ceramides, S1P, and prostaglandins) can modulate the expression or activity of key regulators of SRSPs, including p53, NF-κB, p38 MAPK, JNK, mTOR, IL-1α/β, and STAT3 (173–175). Conversely, some SRSP factors, such as IL-1β, IL-6, IL-8, and TNF-α, can affect lipid metabolism by altering lipogenic enzymes (e.g., FAS, ACLY, and ACC), lipolytic enzymes (e.g., ATGL, hormone-sensitive lipase [HSL], and MGL), lipid transport proteins (e.g., CD36 and FABP4), fatty acid oxidases (e.g., CPT1A, CPT2, and ACADL), and PPAR expression or activity to influence lipid metabolism (173, 176). SRSP factors can also regulate lipids by affecting key signaling pathways such as the phosphatidylinositol 3-kinase (PI3K)/Akt/mTOR and sphingosine kinase 1/S1P pathways.

Disturbances in glucose metabolism can also induce cellular senescence and SRSPs in various tissues, such as pancreatic beta cells, endothelial cells, neurons, astrocytes, myocytes, and adipocytes (177–179). Conversely, SRSPs can modulate its metabolic activity by altering the expression and activity of key metabolic enzymes and transcription factors such as AMPK (180), PGC-1α, sirtuin 1 (181), FoxO (182), NF-κB (183), and nuclear factor erythroid 2-related factor 2 (184). These factors can regulate various aspects of glucose metabolism such as glycolysis, gluconeogenesis, glycogen synthesis and breakdown, pentose phosphate pathway, and the hexosamine biosynthetic pathway. SRSPs can also impair glucose uptake and utilization in these tissues by interfering with insulin signaling and inducing insulin resistance (185).

## 5. Apoptosis, autophagy, and senescence

Apoptosis and autophagy are two major forms of programmed cell death that play important roles in senescence and age-related diseases. Apoptosis is a regulated process of cell elimination that involves the activation of caspases, cleavage of cellular substrates, and formation of apoptotic bodies that are phagocytosed by macrophages or neighboring cells (186). Autophagy is a catabolic process of self-digestion that involves the formation of double-membrane vesicles called autophagosomes, which engulf cytoplasmic components and deliver them to lysosomes for degradation (187). Both apoptosis and autophagy are essential for maintaining cellular homeostasis, tissue integrity, and organismal health, as they remove damaged or redundant cells and organelles, recycle nutrients, and regulate inflammation and immunity (188). However, both processes also decline with senescence, leading to the accumulation of dysfunctional cells and organelles, oxidative stress, chronic inflammation, and impaired tissue repair and regeneration (189).

The relationship between apoptosis and autophagy in senescence is complex and context-dependent. On the one hand, apoptosis and autophagy can cooperate or compensate for each other to maintain cellular quality control and prevent senescence or tumorigenesis. For example, autophagy can remove damaged mitochondria that produce ROS and trigger apoptosis (190). Autophagy can also degrade pro-apoptotic factors or inhibit apoptotic signaling pathways, thus protecting cells from excessive or inappropriate cell death. On the other hand, apoptosis and autophagy can antagonize or compete to modulate cellular fate and function. For instance, apoptosis can inhibit autophagy by cleaving autophagy-related proteins or blocking autophagosome-lysosome fusion. Apoptosis can also induce autophagy as a survival mechanism or a secondary mode of cell death when caspase activation is impaired or overwhelmed (191).

The balance between apoptosis and autophagy in senescence is influenced by various factors, including genetic background, environmental stimuli, hormonal status, metabolic state, and disease conditions. Lipid and glucose metabolism are the two main pathways that provide energy and substrates for cellular function and survival. Dysregulation of lipid or glucose metabolism can affect the balance between apoptosis and autophagy. Lipid metabolism is regulated by various factors, including hormones, nutrients, and oxygen levels. Lipid metabolism can modulate apoptosis and autophagy by affecting the production of ROS, activation of signaling pathways, and formation of lipid droplets or membrane structures (192). For example, excessive lipid accumulation can induce oxidative stress and ER stress, which can trigger apoptosis or autophagy by activating the JNK, p53, or PKR-like endoplasmic reticulum kinase (PERK) pathways (193–195). Conversely, lipid depletion can also induce apoptosis or autophagy by impairing mitochondrial function, reducing ATP levels, or activating the AMPK pathway (196, 197). Moreover, lipid metabolism can influence autophagic membrane initiation and elongation by providing phospholipids or fatty acids as precursors or modulators (198). Lipid metabolism can be regulated by autophagy through the degradation of lipid droplets or lipogenic enzymes in a process called lipophagy (199).

Similarly, glucose metabolism can regulate apoptosis and autophagy by affecting the production of ROS, activation of signaling pathways, and maintenance of energy homeostasis. For example, high glucose levels can induce oxidative stress and ER stress, which can trigger apoptosis or autophagy by activating the p38 MAPK, NF-κB, or CHOP pathways (200–202). Conversely, low glucose levels can also induce apoptosis or autophagy by impairing mitochondrial function, reducing ATP levels, or activating the AMPK pathway (203). Moreover, glucose metabolism can influence autophagy induction and progression by providing hexosamines or acetyl-CoA as regulators (204, 205). Furthermore, glucose metabolism can be regulated by autophagy through the degradation of glycogen granules or glycolytic enzymes in a process called glycophagy (206).

## 6. Insulin signaling

The relationship between insulin signaling and glucose and lipid metabolism is essential for maintaining energy homeostasis and preventing metabolic diseases. Insulin stimulates glucose uptake, utilization, and storage by activating the PI3K/Akt pathway and downstream targets such as GLUT4, glycogen synthase, and HK (207). Insulin also stimulates lipid synthesis and storage by activating the same pathway and downstream targets such as SREBPs, ACC, and FAS (208). Insulin also inhibits glucose production by suppressing the expression and activity of enzymes involved in gluconeogenesis, such as phosphoenolpyruvate carboxykinase (PEPCK) and glucose-6-phosphatase (G6Pase), or lipolysis, such as HSL and ATGL (209). However, insulin signaling and glucose and lipid metabolism can be disrupted by various factors such as obesity, inflammation, oxidative stress, endoplasmic reticulum stress, and senescence. These factors can impair insulin action and induce insulin resistance by interfering with insulin receptor function or downstream signaling components. For instance, oxidative stress can reduce insulin receptor tyrosine phosphorylation and Akt activation by increasing the activity of protein tyrosine phosphatase 1B or protein kinase C (PKC) (210). Inflammation can also impair insulin signaling by inducing the production of pro-inflammatory cytokines such as TNF-α and IL-6, which can activate serine/threonine kinases such as JNK or inhibitor of nuclear factor κB kinase subunit β (IKKβ), that can phosphorylate insulin receptor substrate 1 (IRS-1) on serine residues and inhibit its tyrosine phosphorylation (211). Mitochondrial dysfunction can impair lipid oxidation and increase lipid accumulation in non-adipose tissues such as skeletal muscle or liver, which can cause lipotoxicity and impair insulin action by activating PKC, JNK, or IKKβ (212). Endoplasmic reticulum stress can also induce insulin resistance by activating the unfolded protein response (UPR), which can suppress insulin receptor expression or activate JNK or PERK, which can phosphorylate IRS-1 on serine residues and inhibit its tyrosine phosphorylation (213). Conversely, senescence can also affect insulin signaling and glucose and lipid metabolism by reducing the expression or activity of insulin receptors or their substrates, increasing the levels of inflammatory cytokines or oxidative stress markers, impairing mitochondrial function or autophagy, or altering the composition or function of gut microbiota.

Several transcription factors play pivotal roles in modulating insulin action and lipid homeostasis in response to senescence. For example, FoxO transcription factors, which are downstream targets of the PI3K/Akt pathway, have been implicated in regulating glucose and lipid metabolism, oxidative stress resistance, inflammation, autophagy, and lifespan in various organisms (214). FoxO factors can induce the expression of genes involved in gluconeogenesis such as *PEPCK* and *G6Pase*, or genes involved in FAO such as *CPT1* or *ACOX*, thereby antagonizing the effects of insulin on glucose and lipid metabolism (215). FoxO factors can also induce the expression of genes involved in antioxidant defense such as superoxide dismutase or catalase, or genes involved in anti-inflammatory responses, such as *IL-10* or suppressor of cytokine signaling 3, thereby protecting against oxidative stress and inflammation induced by senescence (216). FoxO factors can also induce the expression of genes involved in autophagy, such as *LC3* or *Atg12*, thereby promoting the clearance of damaged organelles or proteins accumulated during senescence (217). Moreover, FoxO factors can regulate lifespan by modulating the activity of the insulin/insulin-like growth factor signaling (IIS) pathway or other longevity pathways, such as sirtuins or mechanistic/mTOR (218).

## 7. Environmental factors affecting senescence

### 7.1. Nutrition

Glucose is one of the main sources of cellular energy. Excessive glucose intake and abnormally elevated circulating blood glucose levels are associated with chronic diseases such as obesity and diabetes. On the one hand, the metabolic changes caused by hyperglycemia and diabetes promote cellular senescence, leading to tissue dysfunction and various complications such as diabetic retinopathy. Pancreatic β-cells sense elevated blood glucose levels and secrete insulin correspondingly to maintain blood glucose levels within a narrow range. Glycolysis is increased in diabetic β-cells, which inhibits β-cell function (219). Increased numbers of SA-β-gal+ cells in the pancreas of healthy older adults compared with younger adults and further increases in the number of SA-β-gal+ cells in the pancreas of patients with type 2 diabetes (T2D) compared with non-diabetic patients suggest that β-cell senescence may contribute to T2D pathogenesis (220). A diabetic environment and SRSPs are considered drivers of diabetic retinopathy senescence *in vitro* and *in vivo*. High glucose exposure accelerates cellular senescence (20). High concentrations of 25 mM glucose in culture accelerated the senescence of human retinal microvascular endothelial cells. However, glycolysis was significantly reduced rather than increased in an *in vitro* model of senescence. The presence of a negative feedback regulatory mechanism in an *in vitro* model may reduce levels of the glucose transport proteins GLUT1 and GLUT3 and the glycolytic enzyme PFKFB3, affecting the rate of glucose transport (221). On the other hand, cellular senescence may also affect insulin secretion and sensitivity, thereby increasing the risk of developing diabetes. Adipose precursor cells affect neighboring non-senescent cells through the secretion of SRSP factors such as activin A, IL-6, and TNFα, leading to impaired adipogenesis and reduced insulin sensitivity, which may also be involved in T2D progression (222).

Abnormal lipid accumulation during senescence is mainly caused by increased fatty acid uptake, *de novo* lipogenesis, and decreased FAO processes. Mitochondrial integrity and autophagy induction are also diminished during senescence, leading to decreased lipolysis. These changes further impart lipotoxicity to the cell, depleting energy in the tissues and altering cellular signaling, thereby accelerating senescence and the early onset of age-related diseases (223). Multiple adipogenesis enzymes are upregulated in senescent hepatocytes, including FAS, ACC, and stearoyl-CoA desaturase (SCD) (224). In addition to lipid synthesis, excessive lipid intake increases FAO in the mitochondria and the ROS produced, leading to mitochondrial DNA damage and mitochondrial dysfunction (225). Excessive lipid intake inhibits autophagy activation signaling and reduces autophagy levels. Reduced autophagy levels lead to the accumulation of large amounts of waste products and toxins in the cell, interfering with cellular function and homeostasis and accelerating cellular senescence (226). Excess saturated fatty acids activate the NF-κB and JNK signaling pathways by binding to Toll-like receptor 4 on the cell surface, thereby inducing the expression and release of inflammatory factors (227, 228).

### 7.2. Stress

Stress can impair glucose and lipid metabolism by altering the levels of insulin, leptin, cortisol, and other hormones that modulate appetite, energy expenditure, and glucose uptake (229). Stress can also induce lipotoxicity, which is the accumulation of excess lipids and their metabolites in non-adipose tissues, such as the liver, muscle, heart, and brain (230). Lipotoxicity can cause cellular damage, inflammation, insulin resistance, and apoptosis, leading to metabolic disorders and age-related diseases (230). Moreover, stress can affect the mitochondrial function and autophagy of cells, which are involved in glucose and lipid metabolism (231). Mitochondria are organelles that produce energy from glucose and fatty acids, while autophagy is the process that degrades damaged or excess cellular components, such as lipids. Stress can induce mitochondrial stress, which is the imbalance between mitochondrial biogenesis and degradation, resulting in mitochondrial dysfunction, oxidative stress, and impaired energy metabolism (231). Stress can also modulate autophagy, which can have both beneficial and detrimental effects on senescence depending on the stress type and duration. Autophagy can enhance stress resistance and protein homeostasis by removing damaged mitochondria and lipids, but it can also promote cell death and inflammation by activating ferroptosis, a form of iron-dependent lipid peroxidation.

### 7.3. Environment pollution

Environmental pollution, especially air pollution, can expose the body to various harmful substances, such as particulate matter, ozone, nitrogen dioxide, and polycyclic aromatic hydrocarbons. These substances can induce oxidative stress, inflammation, and endothelial dysfunction, which can impair glucose and lipid metabolism (232–234). Oxidative stress can damage DNA, proteins, and lipids, leading to cellular senescence and apoptosis (234). Inflammation can alter the levels of cytokines, such as IL-6 and TNF-α, which can affect the secretion and action of insulin, a hormone that regulates glucose uptake (233).

### 7.4. Temperature

**Figure 3:**
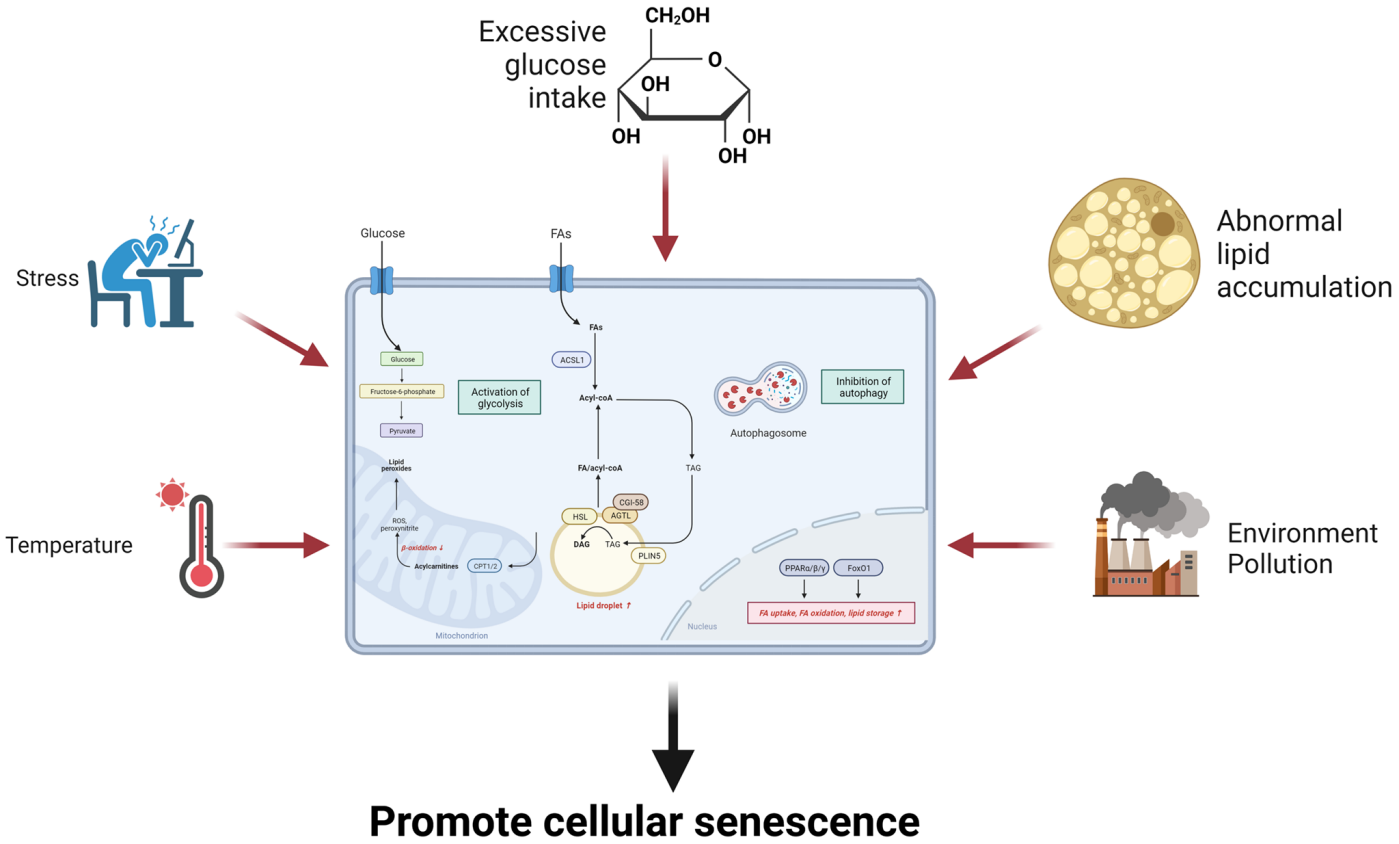
Environmental factors influence senescence through glucose and lipid metabolism. Environmental factors such as excessive glucose intake, lipid overaccumulation, stress, environmental pollution, and temperature can promote cellular senescence by increasing glycolysis and inhibiting fatty acid oxidation.

Glucose and lipid metabolism are regulated by various hormones, enzymes, and signaling pathways that respond to temperature changes, such as thermogenesis, cold exposure, or heat stress (223, 235, 236). Temperature can modulate glucose and lipid metabolism by altering the levels of adipokines, thyroid hormones, catecholamines, and other hormones that modulate energy balance, thermoregulation, and glucose uptake. Temperature can also affect lipid lipolysis and oxidation, which are the processes that break down and utilize lipids for energy. Lipolysis is the hydrolysis of triglycerides into fatty acids and glycerol, while oxidation is the conversion of fatty acids into acetyl-CoA, which is used by the Krebs cycle. Temperature can induce lipolysis and oxidation by activating HSL, ATGL, and CPT, which are enzymes that catalyze lipid breakdown and transport. Temperature can also affect the glycerolipid metabolism and signaling of cells, which are involved in glucose and lipid metabolism (223). Therefore, temperature can influence senescence through multiple aspects of glucose and lipid metabolism that affect cellular health and function (Figure 3).

## 8. Abnormal senescence

### 8.1. Premature ovarian insufficiency

Premature ovarian insufficiency (POI) is the condition of ovarian function decline leading to amenorrhea for over a year before 40 years of age (237), which may be associated with genetic, medical, immunological, environmental, and other factors. POI is associated with metabolic disturbances, including glucose and lipid metabolism, which may increase the risk of long-term complications, such as cardiovascular diseases and osteoporosis (238). Glucose metabolism is regulated by insulin. Estrogen, the main ovarian hormone, modulates insulin sensitivity and glucose homeostasis by influencing insulin secretion, signaling, and action in various tissues. Estrogen deficiency in POI may impair glucose metabolism and lead to insulin resistance, hyperglycemia, or diabetes mellitus (239). Estrogen also affects lipid metabolism by regulating the expression and activity of enzymes and receptors involved in lipid synthesis, transport, and degradation (240). Zhou et al. (238) found that patients with POI had higher levels of plasma glucose, insulin, Homeostatic Model Assessment for Insulin Resistance (HOMA-IR, a marker of insulin resistance), plasma triglycerides, total cholesterol, low-density lipoprotein cholesterol, and apolipoprotein B compared with healthy controls. Moreover, they identified several metabolites related to glucose metabolism and lipid metabolism that were altered in patients with POI, including lactate, pyruvate, citrate, carnitine, acylcarnitine, glycerol, and glycerophospholipids. In conclusion, POI affects not only ovarian function but also metabolic health. POI patients may have impaired glucose and lipid metabolism due to estrogen deficiency, which may increase their risks of developing metabolic disorders or cardiovascular diseases. Therefore, it is important to monitor and manage the metabolic status of POI patients and provide appropriate hormonal replacement therapy or lifestyle interventions to prevent or reduce the adverse outcomes associated with POI.

### 8.2. Platelet senescence

Platelets are cell fragments without nuclei that participate in hemostasis and thrombosis. The lifespan of platelets is usually 7–10 days, and as age increases, the quantity and function of platelets decrease, resulting in increased bleeding propensity (241). The levels of lipid peroxides in platelets increase during senescence. Lipid peroxidation is important for cell function. It causes extensive damage to cell membranes and subcellular granules, leading to enzyme inactivation and destruction, and inhibition of metabolic pathways and cell division. The accumulation of lipid peroxidation products in aged platelets may cause cumulative damage to the membrane structure of platelets and their subcellular granules (such as alpha granules, mitochondria, and the endoplasmic reticulum). Lipid peroxidation may directly affect metabolic pathways by reducing the activity of lipid-dependent enzymes such as NADH- or succinate-cytochrome c reductase or indirectly by releasing lysosomal enzymes from alpha granules (242). Platelets have a rich glycocalyx on their surface, and platelet glycosylation plays an important role in physiological hemostasis mechanisms, regulating platelet-receptor protein interactions, and dynamically remodeling surface glycosylation through their own glucose metabolism system. Platelet glycosylation also participates in platelet senescence and clearance to regulate platelet count; meanwhile, platelet glycosylation abnormalities are closely related to primary immune thrombocytopenia, coronary heart disease, and related diseases, and are potential targets for anti-platelet therapy (243).

### 8.3. Down syndrome

Down syndrome (DS) is a genetic disorder caused by an extra copy of chromosome 21, which results in cognitive impairment, physical abnormalities, and increased risks of age-related comorbidities. Individuals with DS typically exhibit premature senescence phenomena, including skin senescence, graying hair, cataracts, osteoporosis, atherosclerosis, immune decline, and memory loss (244). Likewise, the glucose and lipid metabolism of patients with DS also changes, mainly manifesting as reduced serum cholesterol levels, increased triglyceride levels, increased insulin resistance, abnormal glucose tolerance, etc. These changes may be related to genetic abnormalities, oxidative stress, mitochondrial dysfunction, and other factors in these patients. Moreover, many changes occur in the brain lipids of patients with DS, including a significant reduction in esterified polyunsaturated fatty acids, especially fatty acids esterified into phosphatidylinositol and phosphatidylserine.

## 9. Anti-cancer treatment and senescence

Lipid and glucose metabolism are two essential metabolic pathways that provide energy and building blocks for cancer cells. Cancer cells reprogram their lipid and glucose metabolism to support their rapid growth, survival, invasion, and resistance to therapy.

Cancer cells increase their lipid biosynthesis by upregulating enzymes such as FAS, ACC, and SCD, which are often driven by oncogenic signals such as KRAS, MYC, and PI3K/AKT (245, 246). Cancer cells also enhance their lipid uptake by expressing more lipoprotein receptors, such as low-density lipoprotein receptor (LDLR) and scavenger receptor class B type I (SR-BI), and lipid transporters, such as fatty acid transport proteins and FABPs (245, 247). Moreover, cancer cells can mobilize lipids from adipose tissue or surrounding stromal cells through lipolysis or lipophagy (245, 248). Lipids can be stored in lipid droplets or oxidized through mitochondrial β-oxidation to generate ATP and acetyl-CoA, which can fuel the TCA cycle and oxidative phosphorylation. Lipids can also modulate various signaling pathways, such as RAS, PI3K/AKT, and nuclear receptors, to regulate cell proliferation, survival, migration, and inflammation (245).

In response to this feature, ongoing efforts aim to adapt lipid metabolism as an anti-cancer drug. Some approaches are used in preclinical models *in vitro* and *in vivo*, and some drugs have entered clinical trials. Inhibiting lipid biosynthesis enzymes, such as FAS (TVB-2640) (249), ACC (Soraphen A) (250), and SCD (A939572) (251), can induce endoplasmic reticulum stress and activate the unfolded protein response, leading to cell cycle arrest, apoptosis, or senescence. By blocking lipid uptake receptors like LDLR (GW3965) (252) or SR-BI (ML279) (253), less cholesterol and other lipids are available for membrane production and signaling. This blocking can also impair mitochondrial beta-oxidation and increase the accumulation of toxic lipids, such as ceramide and ROS, by inhibiting the lipid oxidase CPT1 (etomoxir) (254). Inhibition of COX-2 or LPA receptors, lipid signaling molecules, modulates various pathways involved in cancer cell proliferation, survival, migration, angiogenesis, and inflammation (255, 256). Finally, cancer progression can also be inhibited by altering the biophysical properties (fluidity, curvature, and permeability) of the membrane bilayer through the localization and activity of membrane-associated proteins and receptors, as typified by statins that reduce cholesterol concentrations in the plasma membrane and affecting lipid raft clustering and displacement of receptors in non-raft domains (257).

The main pathway of glucose metabolism in cancer cells is aerobic glycolysis, also known as the Warburg effect (258). This is a phenomenon in which cancer cells preferentially convert glucose into lactate, even in the presence of oxygen and functional mitochondria. Aerobic glycolysis allows cancer cells to rapidly consume glucose and produce ATP while avoiding the production of ROS that can damage DNA and proteins. Moreover, aerobic glycolysis provides cancer cells with various metabolic intermediates that can be used for the synthesis of nucleotides, amino acids, and lipids (259). To support aerobic glycolysis, cancer cells increase their glucose uptake by overexpressing GLUTs, especially GLUT1 (260). Cancer cells also upregulate key glycolytic enzymes, such as HK2, phosphofructokinase 1, pyruvate kinase M2, and LDHA, which are often regulated by oncogenic signals, such as KRAS, MYC, and HIF1α (261). However, aerobic glycolysis is not the only mode of glucose metabolism in cancer cells. Some cancer cells can also use oxidative phosphorylation (OXPHOS) to generate ATP from glucose-derived pyruvate in the mitochondria. OXPHOS can be activated in cancer cells under conditions, such as hypoxia, nutrient deprivation, or drug resistance (262). OXPHOS is more efficient than glycolysis in terms of ATP production per glucose molecule but also generates more ROS. OXPHOS can also provide cancer cells with acetyl-CoA and NADH, which can modulate various signaling pathways, such as histone acetylation and sirtuin activity. To support OXPHOS, cancer cells can modulate their pyruvate metabolism by regulating the expression or activity of pyruvate dehydrogenase, pyruvate dehydrogenase kinase, or pyruvate carboxylase. Cancer cells can also use alternative substrates for OXPHOS, such as glutamine, fatty acids, or ketone bodies (261).

Cellular senescence can occur in both normal and cancer cells and has complex dependence effects on cancer development and treatment. On the one hand, cellular senescence is a cancer suppressor mechanism that prevents the proliferation of damaged or malignant cells. SRSPs secreted by senescent cells recruit and activate immune cells to remove senescent cells (263). Some immune cells, such as T helper 1 cells, can also trigger cancer cell senescence by secreting inflammatory cytokines. In addition, cellular senescence enhances the expression of cancer-associated antigens and immunogenic molecules on cancer cells, making them more susceptible to recognition and clearance by T cells. Thus, T cells are a key component of cancer immunotherapy (263). On the other hand, cellular senescence can also adversely affect T cell immunity and cancer therapy. First, senescent cells accumulate in cancers and normal tissues, releasing SRSPs with pro-tumorigenic effects such as promoting angiogenesis, invasion, metastasis, and drug resistance. Second, T cell senescence decreases their proliferation, cytokine production, and cytotoxicity and increases the expression of inhibitory receptors and pro-apoptotic molecules (264, 265).

Therefore, cellular senescence is a double-edged sword that can influence T cell immunity and cancer therapy in different ways. Understanding the molecular mechanisms and interactions between how sugar and lipid metabolism regulate T cell senescence could provide new insights and strategies for improving anti-cancer therapy. T cell metabolism is tightly linked to T cell activation, differentiation, and function. T cells undergo metabolic reprogramming upon antigen stimulation, switching from a quiescent state that relies on oxidative phosphorylation to a highly glycolytic state that supports rapid proliferation and effector functions (173). However, chronic or excessive stimulation can lead to T cell exhaustion or senescence, which are associated with metabolic dysregulation, such as impaired glucose uptake, reduced glycolytic capacity, and altered mitochondrial function (266). In addition to glucose metabolism, lipid metabolism plays a crucial role in regulating T cell immunity. Senescent T cells have reduced FAO capacity and increased dependence on FAS. Targeting FAS with pharmacological inhibitors or gene knockdown induces apoptosis in senescent non-functional T cells and improves anti-cancer immunity. Conversely, enhancing FAO with pharmacological activators or gene overexpression can prevent or reverse T cell senescence and exhaustion by restoring mitochondrial function and reducing oxidative stress (267).

## 10. Conclusion

The relationships between different types of lipid components, glucose metabolites, and senescence have been reported (268, 269). However, an in-depth study of the regulatory relationships of the key enzymes is necessary to screen anti-senescence drugs. New anti-cancer drugs may also be developed by targeting tumor cell senescence.

**Figure 4:**
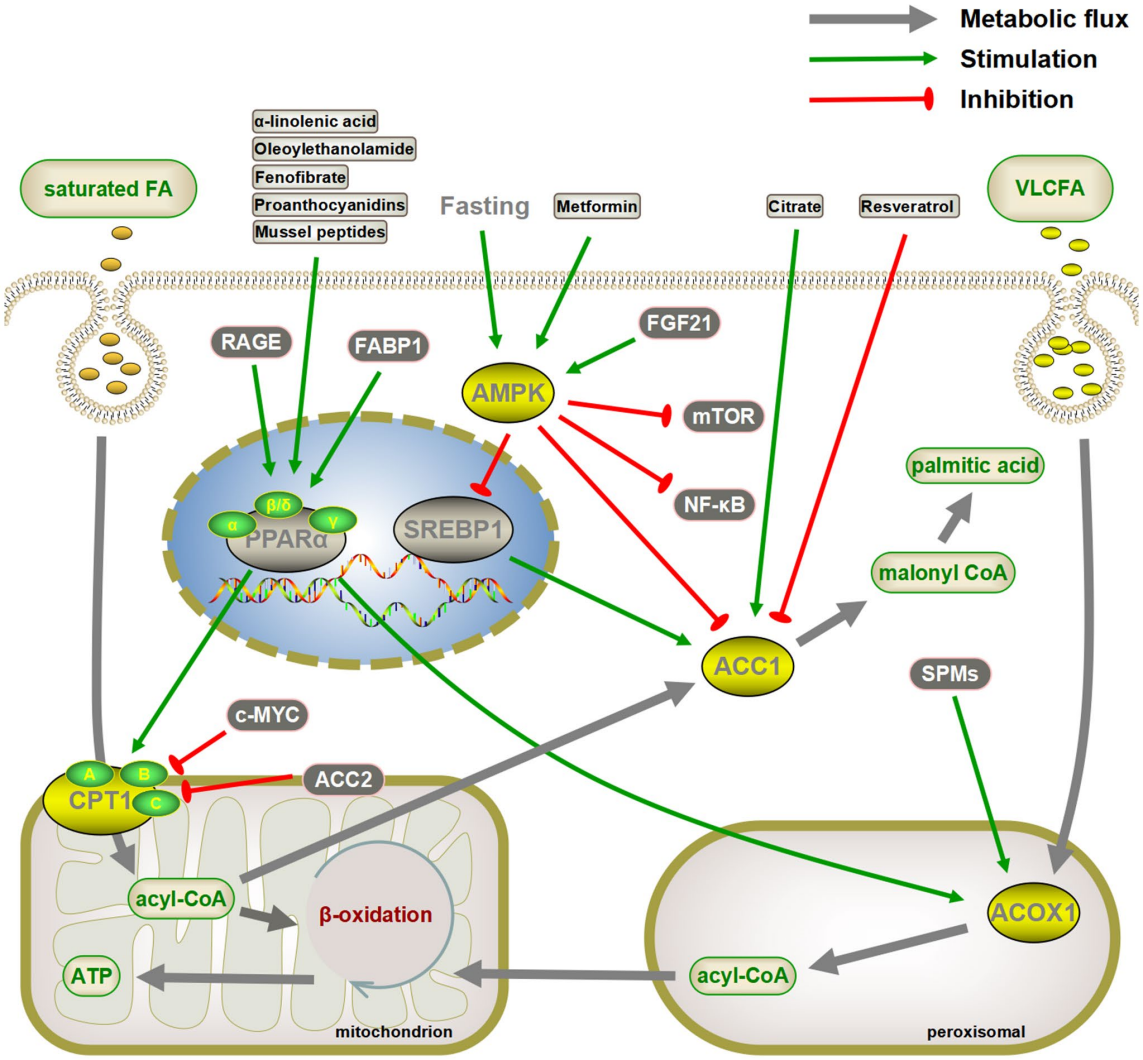
Targeting critical enzymes of fatty acid synthesis and catabolism to delay senescence. Activation of CPT1 and ACOX1 directly or indirectly through the peroxisome proliferator-activated receptor α (PPARα) pathway promotes fatty acid oxidation, both of which may have a senescence-delaying effect. Similarly, inhibiting fatty acid synthesis by the AMPK/SREBP1/ACC pathway is also beneficial in slowing down senescence. AMPK also inhibits senescence by inhibiting the mTOR and NF-κB pathways. The gray arrows represent normal metabolic pathways. The green pointed and blunt red arrows represent activating and inhibiting effects, respectively.

**Figure 5:**
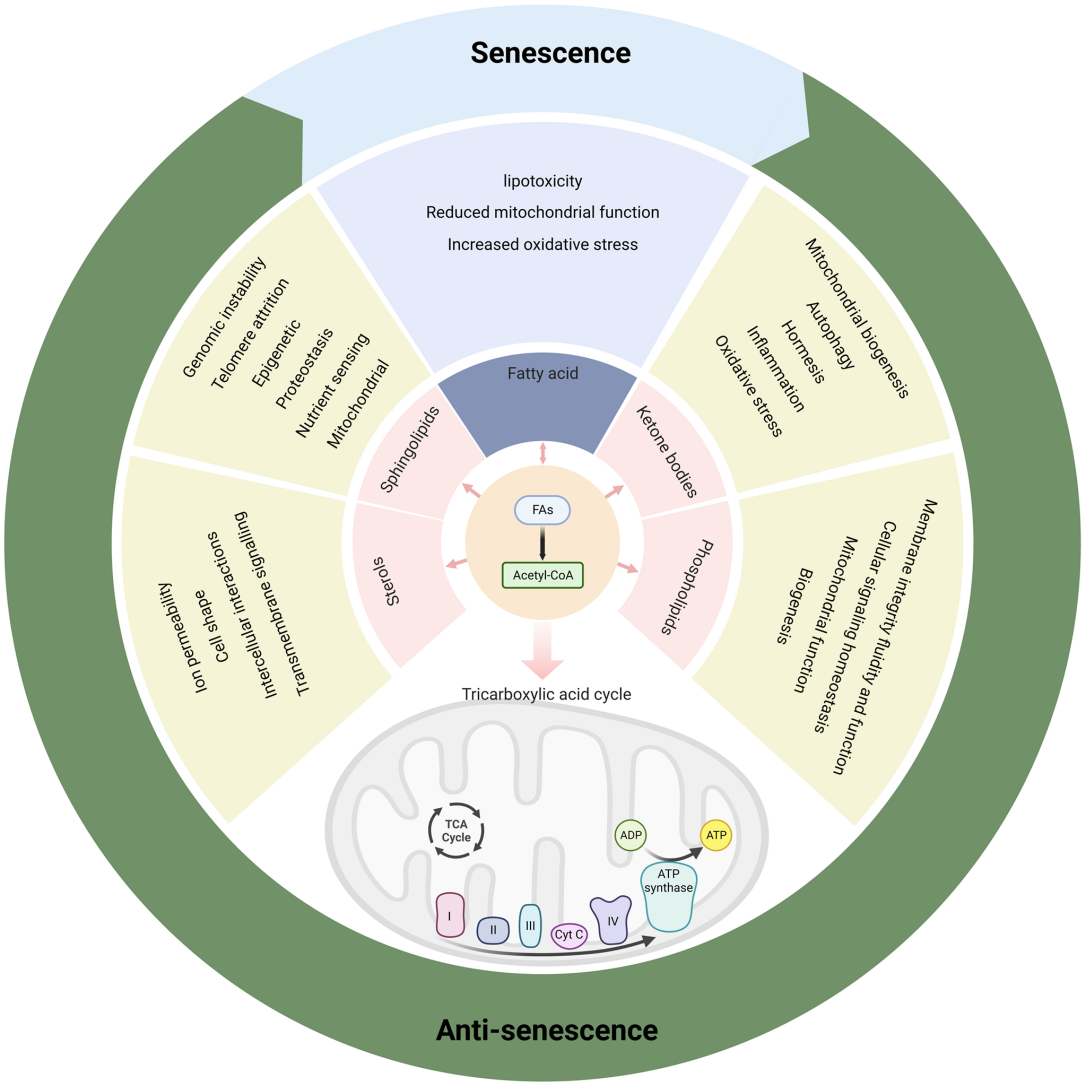
Various metabolic products of fatty acids have rich biological functions and affect senescence.

Various aspects of glucose and lipid metabolism are closely related to senescence. In lipid metabolism, decreased fatty acid catabolism is directly related to senescence onset, including mitochondrial β-oxidation (34, 36, 37, 57, 270), peroxisomal β-oxidation (54, 55), α-oxidation (82, 84), and ω-oxidation (85). The most prominent key enzymes include CPT1 and ACOX1. Drugs such as fenofibrate can exert anti-senescence effects by targeting PPARα, the gene upstream of *CPT1* and *ACOX1* (Figure 4). Meanwhile, increased fatty acid synthesis aggravates lipotoxicity, and both direct inhibition of ACC and indirect regulation via AMPK/SREBP1 can exert anti-senescence activity (89). In addition to fatty acid catabolism and synthesis, acetyl coenzyme A undergoes a variety of pathways to ketone bodies, phospholipids, sphingolipids, and sterols, which perform a wide range of biological functions and exert pro- or anti-senescence effects (Figure 5). The effects of these lipids on senescence often depend on the amount, subtype, and state of the cellular microenvironment. For example, appropriate levels of ketone bodies are beneficial for the central nervous system’s energy supply, maintenance of mitochondrial biogenesis, and attenuation of oxidative stress; however, excessive ketone bodies can lead to ketoacidosis and even death (118). Cer induces cellular senescence, whereas S1P, another sphingolipid component, delays senescence and extends lifespans (133, 137).

**Figure 6:**
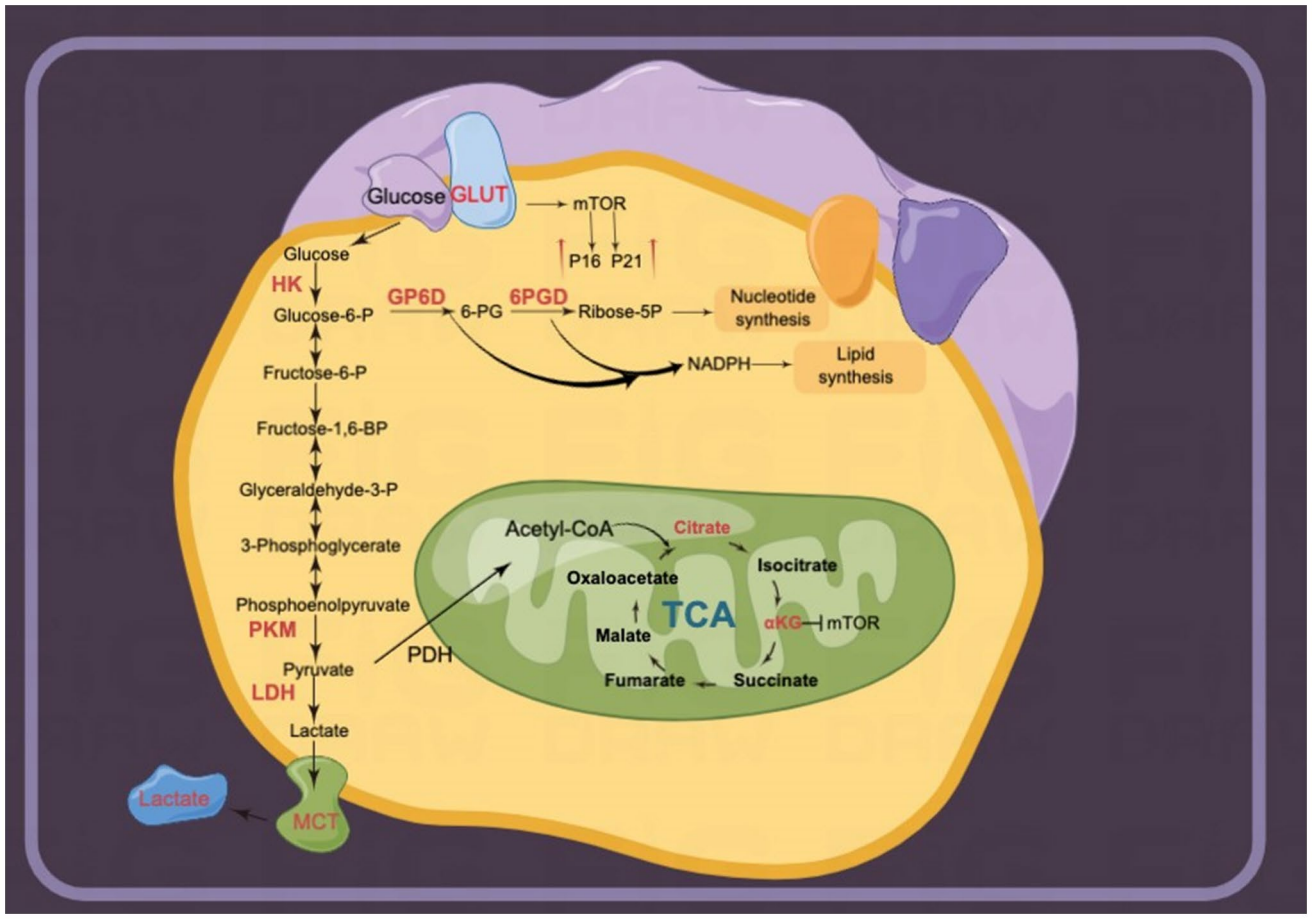
Glucose metabolism is upregulated in senescent cells. Multiple enzymes of glycolysis are upregulated. For instance, lactate release mediated by monocarboxylate transporters (MCTs) is involved in senescence-related secretory phenotypes (SRSPs). Several metabolites in the tricarboxylic acid (TCA) cycle, including α-ketoglutarate (αKG) and citrate, delay senescence.

Multiple glucose metabolism pathways are involved in the regulation of intracellular senescence. Several rate-limiting enzymes in glycolysis, including HK2 and PK, are highly expressed in senescent cells (150, 156). Although the pharmacological activation of Nrf2 increases HK2 expression (18), other mechanisms require further investigation. Several key enzymes of the PPP, a branch of the glycolytic pathway, are also involved in the regulation of intracellular senescence. The key enzymes promote cellular senescence by upregulating P21, P16, and telomerase activity (163). Notably, the dietary TCA-cycling metabolites citrate and αKG delay cellular senescence (23–26). Therefore, supplementation of these metabolites may provide an effective intervention for the treatment of senescence-related dysfunction (Figure 6).

Environmental factors can affect senescence through glucose and lipid metabolism, which are essential for energy production and cellular function. Alterations in the external environment such as nutrition, stress, environment, pollution, and temperature accelerate cellular senescence by regulating glucose and lipid metabolism through a variety of hormones, enzymes, and signaling pathways. Thus, targeting aberrant metabolism may delay normal cellular senescence.

In summary, fatty acid and glucose metabolism are essential in the maintenance of normal cellular function, both of which are disturbed by senescence. Conversely, disturbances in fatty acid and glucose metabolism can further lead to senescence. Two pathway-related enzymes and metabolites are involved in the regulation of cellular senescence. While many advances have been made in recent years, several important issues must still be addressed, including: (a) What are the initiating factors of senescence and metabolic disorders? (b) While reports suggest a correlation between metabolic disorders and senescence, the true causal relationship remains undetermined. (c) Anti-senescence therapies proven to work in humans are scarce. Understanding the role of enzymes and metabolites in fatty acid and glucose metabolism may provide answers for a more comprehensive understanding of senescence.

## Author contributions

BL and QM investigated and wrote the first draft of the manuscript. XG and HS wrote sections of the manuscript. ZX and YW contributed to conception the study and acquire funds. HZ contributed to design of the study and acquire funds. BL, QM, XG, HS, ZX, YW, and HZ were involved in drafting, revising the manuscript, and agree to be accountable for the content of the work. All authors contributed to the article and approved the submitted version.

## Funding

This work was supported by the National Natural Science Foundation of China (Nos.: 82270785 and 82020108024) and Science and Technology Department of Jilin Province (No. 20230101138JC).

## Conflict of interest

The authors declare that the research was conducted in the absence of any commercial or financial relationships that could be construed as a potential conflict of interest.

## Publisher’s note

All claims expressed in this article are solely those of the authors and do not necessarily represent those of their affiliated organizations, or those of the publisher, the editors and the reviewers. Any product that may be evaluated in this article, or claim that may be made by its manufacturer, is not guaranteed or endorsed by the publisher.
